# Balanced fertigation and improved sustainability of June bearing strawberry cultivated three years in open polytunnel

**DOI:** 10.3233/JBR-170157

**Published:** 2017-08-18

**Authors:** Rolf Nestb, Sébastien Guéry

**Affiliations:** a Norwegian Institute of Bioeconomy Research (NIBIO), Ås, Norway; b Optiriego Consulting, Edificio CREA modulo n°5, C. José Galán Merino SN, Sevilla, Spain

**Keywords:** Cultivar, fruit yield, ions, NBI, water, soil depth, leaching

## Abstract

**OBJECT:**

Improved precision fertilization by introducing sensors and remote control to secure fruit yield and reduce nutrient leaching in soil culture.

**MATERIAL AND METHODS:**

We broadcasted before bedding and mulching 50 g m^–2^ of a multi-mineral fertilizer. Beds had two plant rows 20 cm apart, with plant distance of 25 cm. Experimental design was split plot with three replications and three treatments. Treatments: fertigation in large plots, cultivar in small plots and year.

**RESULTS:**

Plant development in the establishing year had no benefit of fertigation in addition to fertilizer given before bedding. When the yield is 3 kg m^–2^ a nutrient solution of 6 g N m^–2^ gave highest yield, using 4 g m^–2^ from two weeks before harvest and during harvest. ‘Florence’ and ‘Sonata’ developed well; however, ‘Florence’ had mildew on fruits in the last cropping year. ‘Korona’ presented well the first cropping year, but grew small fruits heavily infested by mildew in the last cropping year.

**CONCLUSION:**

Fertilization had effect on fruit yield. It is discussed how a fertilization schedule for the establishment year and cropping years can be adapted to plant development stages. Mildew infestation on fruits was dependent of cultivar and fertilization. Introducing sensors for recording of growth factors and *in situ* ion-levels of soil water nutrients, proved valuable.

## 1 Introduction

Strawberry growing in Norway is mainly extensive cultivation of June bearing cultivars on matted row on low beds and to some extent double row on polyethylene mulched high beds. Growers give granulated fertilizers before planting, a practice supported by research [1, 2]. However, it is shown that broadcasted NO_3_-N given in April/May and late August of the harvest years increased fruit yield of ‘Bounty’ compared with none or one fertilization incidence [3]. NO_3_-N fertigation given only in May and August increased fruit yield of ‘Korona’ and ‘Bounty’ in the second crop year on both a silt loam and on a silt clay, but in the first fruiting year only on the silt loam [4]. The practice of combined spring and autumn fertilization is still a recommended standard in Norway. On the other hand, introducing drip irrigation on high beds and growing in high polyethylene tunnels on agricultural land has made it possible to add fertilizer in the drip-water (fertigation), narrowing the gap to soilless culture. This has gradually changed the use of fertilizer from relative slow release multipurpose fertilizers to easily dissolved fertilizers. The uptake of macro- and micro-elements in ‘Elsanta’ at different growth stages in soilless culture, was investigated to improve the knowledge of uptake of nutrients to different organs [5]. This knowledge is valuable also for growing in soil. With relative poor knowledge of how to use easily dissolved fertilizers in soil culture, originally meant for soilless culture, over-fertilization frequently occurs. This may cause replant failure and increased salt accumulation in soils and ground water contamination [6]. Increased knowledge of nutrient uptake in strawberry together with new tools to manage fertigation opens a window for more precise fertigation. The problem of loss of NO_3_-N by leaching could by combining precise irrigation and moderate N fertigation be minimized [7]. It was proposed to develop a nutrient formulation according to ratio of mineral nutrients absorbed by the crop; managing amount of N fertilizer required for ensuring the target growth by controlling EC of the nutrient solution, applying macro nutrients on the basis of their specific ratios to N [8]. Since NH4 is an important N-source in cold weather a mixture with 80% NO_3_ and 20% NH_4_ was recommended [8, 9]. However, to adjust the fertigation quickly, *in situ* methods are necessary to address the nutrient situation at all growth stages. This is possible by using sensor technology that are available by severalcompanies.

Recently, Norwegian growers have expressed the need of more information on how to fertilize and how to control water, which initiated this study. We have controlled water level in soil, added fertilizers in concentrations adapted to strawberry [5], sampled soil water for analyzes of nitrate- and ammonium-N and macro nutrients using an *in situ* method. Additionally was the leaf Nitrogen Balance Index (NBI) recorded to find if it could be a corrector to N-fertigation as suggested by others [10, 11].

## 2 Material and methods

The field trial was on a silt clay in open high polyethylene tunnel (polytunnel). The pH was 6.0 and content of macro mineral nutrients was 11.0, 8.9, 13,5 and 216 for P_AL_, K_AL_ Mg_AL_ and Ca_AL,_ respectively; which was on the high side for all except K that was lower than recommended level, however available K was relatively high (K_HNO3_ = 165). To adjust the level of macro nutrients in soil to strawberry culture, we fertilized with 50 g m^–2^ of a multi mineral fertilizer (YaraMila Fullgjødsel 12-4-18 micro™, YARA Norway) before bedding and mulching, which is standard recommendation for a silt clay in the region. Beds were 20 cm high, 60 cm wide and the distance between bed centers was 150 cm. We mulched the beds with brown polyethylene. Two rows planted on each bed 20 June 2013 were 20 cm apart, with 25 cm plant distance within row. The plants were overwintered plug plants of ‘Korona’, ‘Sonata’ and ‘Florence’ from a local nursery.

### 2.1 Experimental design

The experimental design was split-plot with three replications. Treatments were A) fertilization (mix of YaraLiva™ Calcinit and Kristalon™ Indigo, YARA Norge AS, Norway) on large plots, In the planting year (2013) fertilization levels were, I: Control (tap water EC≈0.1), II: Fertilizer equivalent to 1 g N m^–2^ and III: Fertilizer equivalent to 3 g N m^–2^. In each of the two cropping years fertilization levels I, II and III were 0, 3 and 6 g N m^–2^, respectively; B) cultivar (‘Korona’, ‘Sonata’, ‘Florence’) on small plots and C) year (2013, 2014, 2015). Treatments A and B were randomly distributed within block, respectively between large and small plots. Each small plot was 3.75 m long (30 plants) and the harvest plot contained 12 plants.

### 2.2 Technical approaches to secure correct fertigation practice

To control the amount of fertilizer given to each bed (large plot) within the block, each drip pipe of the block was equipped with a water meter to control amount of nutrient solution given during a certain amount of time. The water situation of the soil was registered using EM50G loggers (Decagon Devices, USA) equipped with EC-5 sensors (Decagon Devices, USA) for measuring volumetric water content in soil at 10, 20 and 30 cm depths, at each fertilization level (large plot). One logger was equipped with three multiple GS3 sensors (Decagon Devices, USA) at the same depths as the EC-5 sensors, which each, in addition to volumetric water content, register soil temperature and bulk electro-conductivity. We established a “Full Stop Wetting Front Detector” (CSIRO, Australia) to collect soil-water at 15 and 25 cm depths on small plots of ‘Sonata’ [12]. At 18 July 2013 the establishment of all EM50G loggers with sensors and “Full Stop” devices wasfinished.

We used two nutrient stock solutions (7.5 kg YaraLiva™ Calcinit per 100 l of water and 7.5 kg per l of Kristalon™ Indigo per 100 l of water) in the establishing year, and in May and June in the cropping years. In July and later in the two cropping years, we changed the solution of Kristalon™ Indigo to 9.0 kg per 100 l of water to balance the nutrient solution to a generative plant development stage. The fertilizer was given through drip pipes (fertigation) once a week using an injector (D3GL2, DOSATRON^®^, France), with a given amount of N per minute. It was injected tap water in the two first minutes, after that fertilizer was added the necessary minutes dependent on the fertilization treatment, and subsequently followed by tap water to make the total time span of each treatment equal concerning the volume sum of water. We supplied control with tap water as long as for fertigation. Besides, the field was drip-watered when amount of plant available water at 20 cm depth went below 70% of plant available water compared to field capacity (100%).

### 2.3 Sampling

We sampled soil-water from the “Full Stop” half an hour after fertigation. The samples were frozen at each sampling date, and analyzed later after thawing and adjustment to room temperature for NO_3-_, NH^4+^, Ca^2+^, K+, Mg^2+^ and Na^+^, using a handheld multi-ion meter (CG001, CleanGrow, UK).

We registered total and marketable fruit yield in g per plot, and sorted marketable fruit yield into the fruit size classes: ≥35 mm, 30–35 mm, 25–30 mm, 22–25 mm and ≤22 mm in diameter. Not marketable fruit was rotten, misshapen and rest (mildew, birds, snail damage, etc.) recorded in g per plot. Additionally, flavonoid level (Fl) in μg cm^–2^ and chlorophyll level (Chl) in μg cm^–2^ were recorded, and NBI = Chl/Fl was calculated as average of measurements on the abaxial and adaxial sides of ten leaves from each small plot in the two harvest years, using the DUALEX™ Scientific sensor (Force A, France). In spring 2015 we made a 35 cm deep bed profile in a ‘Sonata’ plot of fertilization level II using a sharp spade and photographed the profile.

Statistics were undertaken using the SAS procedures GLM, tabulate and graph [13].

## 3 Results and discussion

### 3.1 Effect of fertilization

#### 3.1.1 Fruit yield

There was no interaction concerning total fruit yield or marketable fruit yield, neither for fertilization vs year nor for fertilization vs cultivar, and therefore is fruit yield presented as average of cultivars (Table 1). Considering the two-year mean, the level of fertilizer had effect on total fruit yield. The highest fertilizer level (III) improved yield compared with level II and control, but fruit yield at level II was not significantly different to control. However, there was no significant differences between marketable yields in average of years. Fruit weight increased by fertilization. The percentage of small fruits was highest in control in average of the two years. Percentage of misshaped fruits and rest was not significantly different between fertilization levels.

**Table 1:** Influence of three fertigation levels (FL) on fruit weight (Fw) in g fruit^–1^, total- and marketable- fruit yieldin g m^–2^ and percentage fruit yield (%) of fruits equal to or smaller than 25 mm diameter (Sf), misshapenfruit (Mf) and rest, in two harvest years as average of three strawberry cultivars

FL	Year	Fruit yield			%		
		Fw	Total	Market	Sf	Mf	Rest
Control	2014	15.1	1.448	1.378	3.79	0	4.9
II		16.5	1.474	1.416	2.74	0	4.0
III		15.5	1.782	1.709	4.16	0	4.1
Mean		15.7	1.568	1.501	3.71	0	4.3
Se		1.1^ns^	0.250^ns^	0.25^ns^	0.79^ns^	0	0.6^ns^
Control	2015	16.1	2.771	2.397	8.70	1.91	11.4
II		17.6	3.098	2.611	7.16	0.91	14.0
III		17.6	3.636	2.852	5.07	0.77	23.6
Mean		17.1	3.150	2.611	6.98	1.21	16.3
Se		1.0^ns^	0.354^*^	0.370^ns^	0.53^**^	0.41^**^	0.9^**^
Control	Mean	15.6	2.109	1.888	6.23	0.96	8.1
II		17.0	2.286	2.013	4.96	0.46	9.1
III		16.5	2.655	2.247	4.62	0.39	13.5
Mean		16.4	2.350	2.049	5.27	0.61	10.2
Se		0.71^*^	0.216^*^	0.225^ns^	0.81^*^	0.24^ns^	3.8^ns^

^*,**,***,ns^indicate significant differences, respectively at levels of 5%, 1%, 0.1% and no significance.

Results varied between years. In 2014 (first crop year) there was no significant effect of fertilization neither for total nor marketable yield. This is comparable to results achieved in the first harvesting year for ‘Korona’ and ‘Bounty’ grown in open field at the same location and on the same soil type as here, fertilized with a nutrient solution of Calcinit (Yara, Norway) [14]. There were no misshapen fruits that year, and fruit weight equal to or smaller than 25 mm diameter (Sf) and rest was unaffected by fertigation. There was no significant effect on fruit size by fertilizer.

In the second crop year strongest fertilization increased total fruit yield compared to control. However, marketable fruit increased but not significantly; the main reason was the strong infestation of mildew on ‘Korona’ and to some extent on ’Florence’, at strongest fertilization, which dominated the rest portion. Fertilization had no significant effect on fruit weight, but fruit weight was higher than in 2014; in spite of higher yields than the year before. The differences in yield between the years could partly be explained by the much warmer September and October in 2014 (T_average_ 9.1°C) than in 2013 (T_average_ 5.0°C), that provided better conditions for flower bud development in the autumn of 2014 and favored high fruit yield and fruit size in 2015. The percentage of small and misshapen fruits was lower for fertilized plants than for control in 2015.

#### 3.1.2 Soil ion concentration

The concentration of ions in soil water changed throughout the three seasons, as a reflection of fertilization that increased during the establishing season parallel to increasing plant size, and in the two crop seasons because of vegetative development and fruit ripening (Fig. 2, Tables 2–4). Since strawberry is sensitive to salinity [15, 16], we suggest that the species should be in saline class weak of Wolf [15]. That imply EC-levels in soil (1soil:2water) from 0.51 to 1.50 mS cm^–1^, where the lower part of the scale may indicate too little N and K for rapid growth [16], which also are in agreement with practice in present Spanish strawberry growing. The ion values are from soil water sampled the same day as fertigation took place. We fertilized at weekly intervals on the same week-day and at the same time of the day. The level of ions would drop between one fertigation and the next, depending on the level of watering between the days of fertigation (not tabulated), and uptake by plants.

**Fig.1:**
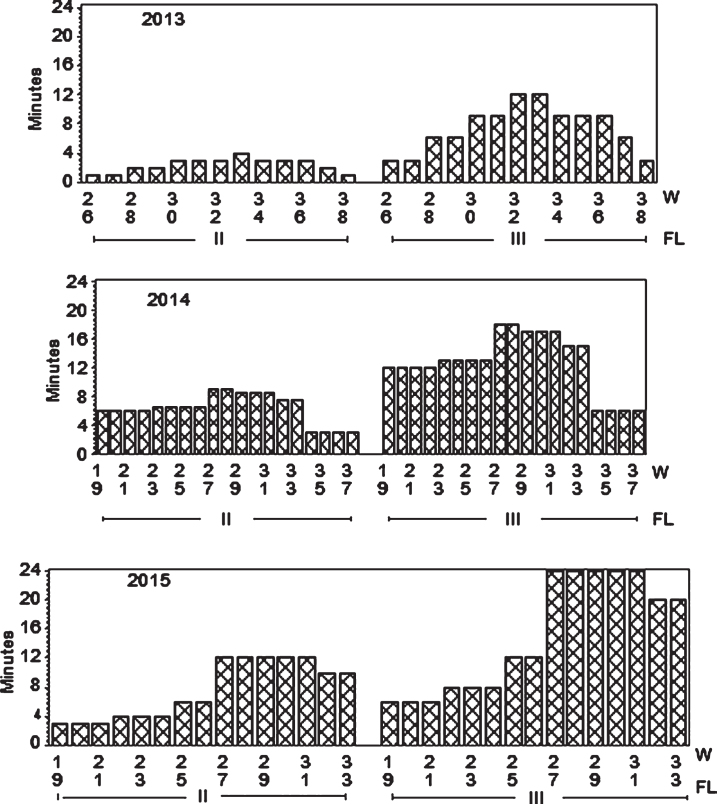
Program showing weekly fertigation in minutes for two fertilization levels (FL II and III = 1 and 3 g of N m^–2^, respectively, in 2013; and 3 and 6 g N m^–2,^respectively in 2014 and 2015. The third level Control (I) = 0 fertilizer is not shown.

**Fig.2:**
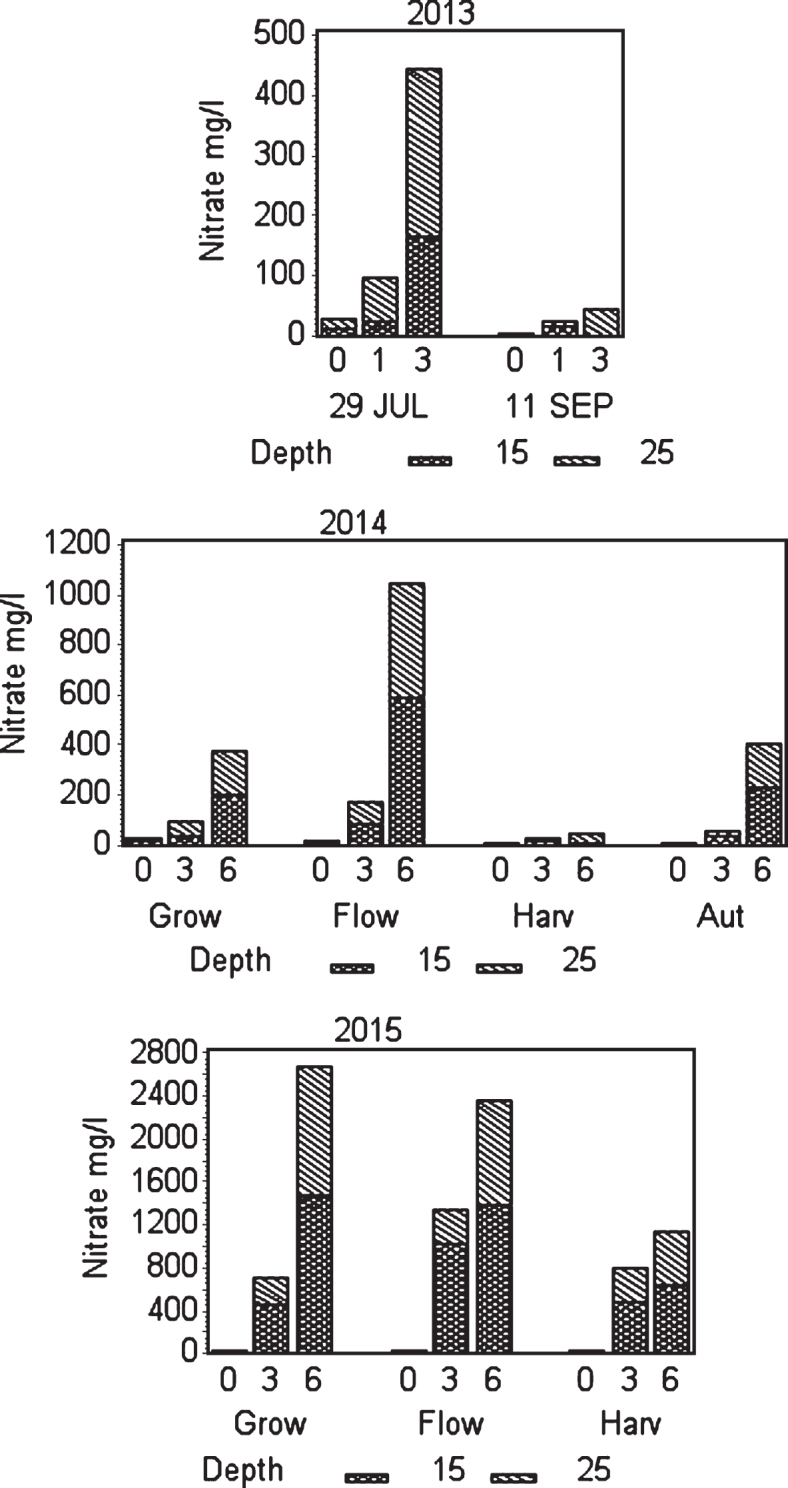
Effect of fertilization on NO_3 ÷_ concentration in soil water at two depths. In 2013 the sampling was taken at one date in July and one in September. In 2014 and 2015 the average of all samples in the the period before flowering (Grow), during flowering (Flow), during harvest (Harv) and after harvest (Aut) is shown. In 2015 fertilization stopped after harvest.

**Table 2:** Influence of three fertigation levels (FL: Control = 0, II = 1 g N m^–2^ and III = 3 g N m^–2^) on ionconcentration in mg l^–1^ and electric conductivity in mS cm^–1^ (EC) of soil water. At two depthsin the year of planting (2013) and at two dates for ‘Sonata’ grown in open polytunnel

Date	FL	Depth cm	Ion concentration			EC
			Ca^2+^	Mg^2+^	K^+^
29.07	Control	15	39	1.4	6.0	0.21
		25	45	2.0	6.0	0.19
	II	15	32	1.3	5.6	0.16
		25	59	2.5	6.1	0.24
	III	15	111	6.2	17.7	0.33
		25	156	7.9	17.8	0.35
11.09	Control	15	31	2.8	2.2	0.18
		25	33	3.0	2.4	0.17
	II	15	26	1.9	3.0	0.18
		25	37	2.6	3.4	0.24
	III	15	40	3.5	8.0	0.28
		25	46	4.1	8.6	0.26
Mean		15	47	2.9	7.1	0.22
		25	63	3.7	7.4	0.24
Se			30^*^	1.4*	1.9^***^	0.02^**^

^*,**,***,ns^indicate significant differences, respectively at levels of 5%, 1%, 0.1% and no significance.

**Table 3:** Influence of three fertigation levels (FL: Control = 0, II = 3 g N m^–2^ and III = 6 g N m^–2^) on ionconcentration in mg l^–1^ and electric conductivity in mS cm^–1^ (EC) of soil water. At two depths in cm,at four growth stages (GS) in the first year of harvest (2014), for ‘Sonata’ grown in open polytunnel

			Ion levels			EC
Gs	FL	Depth	Ca^2+^	Mg^2+^	K^+^	
Growth	Control	15	18	1.0	2.4	0.19
		25	19	0.9	1.1	0.18
	II	15	16	0.8	2.6	0.18
		25	20	0.9	1.6	0.20
	III	15	27	1.2	2.9	0.26
		25	42	1.5	5.1	0.34
Flower	Control	15	9	0.7	0.9	0.08
		25	8	0.7	0.5	0.08
	II	15	25	2.0	6.7	0.22
		25	30	2.2	2.7	0.28
	III	15	137	12.7	43.7	0.96
		25	111	8.2	23.3	0.74
Harvest	Control	15	21	2.2	0.4	0.11
		25	19	2.2	1.2	0.11
	II	15	14	2.4	4.2	0.17
		25	20	3.0	2.5	0.17
	III	15	31	5.1	11.2	0.24
		25	36	5.8	9.0	0.25
P.harv	Control	15	20	2.5	0.5	0.11
		25	14	2.3	0.3	0.08
	II	15	20	3.5	1.5	0.19
		25	18	2.9	1.2	0.09
	III	15	115	16.0	24.5	0.81
		25	86	10.9	11.2	0.59
Mean			37	3.8	6.7	0.28
Se			2.4^***^	0.4^***^	1.4^***^	0.02^***^

^*,**,***,ns^indicate significant differences, respectively at levels of 5%, 1%, 0.1% and no significance.

**Table 4:** Influence of three fertigation levels (FL: Control = 0, II = 3 g N m^–2^ and III = 6 g N m^–2^) on ion concentration in mg l^–1^ and electric conductivity in mS cm^–1^ (EC) of soil water. At two depths in cm, at three growth stages (GS) in the second year of harvest (2015), for ‘Sonata’ grown in open polytunnel

Gs	FL	Depth	Ion levels			EC
			Ca^2+^	Mg^2+^	K^+^
Growth	Control	15	11	1.5	0.8	0.09
		25	11	1.6	0.6	0.07
	II	15	128	16.3	18.7	0.70
		25	81	8.8	5.9	0.53
	III	15	508	60.0	93.4	2.75
		25	359	30.8	33.9	1.86
Flower	Control	15	4	0.8	0.6	0.10
		25	3	0.7	0.3	0.04
	II	15	51	8.6	15.5	0.49
		25	41	6.4	4.4	0.46
	III	15	229	33.3	81.3	1.61
		25	216	25.0	34.1	1.40
Harvest	Control	15	4	0.9	0.5	0.09
		25	5	1.0	0.4	0.07
	II	15	10	2.3	4.7	0.34
		25	33	5.0	6.0	0.34
	III	15	93	13.3	38.3	0.80
		25	71	9.2	20.1	0.63
Mean			103	12.7	19.7	0.66
Se			12.8^***^	1.5^***^	2.9^***^	0.07^***^

^*,**,***,ns^indicate significant differences, respectively at levels of 5%, 1%, 0.1% and no significance.

July 29th 2013 was the first soil water sampled. Results show that concentration of macro nutrient cations and EC recorded was higher at fertigation level III (3 g N m^–2^) than at control and level II (1 g N m^–2^). The difference between control and fertilizer level II was not significant for any parameter except EC, which was lower for level II than for control at 15 cm depth and higher at 25 cm depth (Table 2). Also, NO_3_^–^ concentration was higher at fertigation level III compared with control and fertilization level II; but not too high since the EC value was relatively low (Fig. 2, Table 2). The samples from 11 September 2013, showed Ca–, Mg–and K–ion values with not much difference for control and fertigation level II (Table 2); the concentrations of nitrate were relatively low (Fig. 2). However, at strongest fertigation the content of cations increased; and the concentrations of nitrate increased at 25 cm depth compared to 15 cm depth. EC level at highest fertigation was low and do not indicate to high use of fertilizer. However, the results indicate movement of excess nitrate downwards in the soil profile in summer and autumn, and similar for calcium in summer; probably because of too long fertigation pulses.

**Fig.3:**
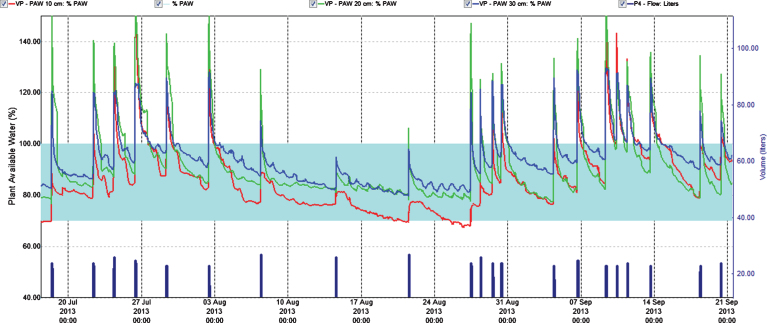
Percentage plant available water at three soil depths (Red = 10 cm, Green = 20 cm, Blue = 30 cm) in the establishing year (2013). Easily available water (70–100%) is within the turkis zone. 100% available water = FC.

**Fig.4:**
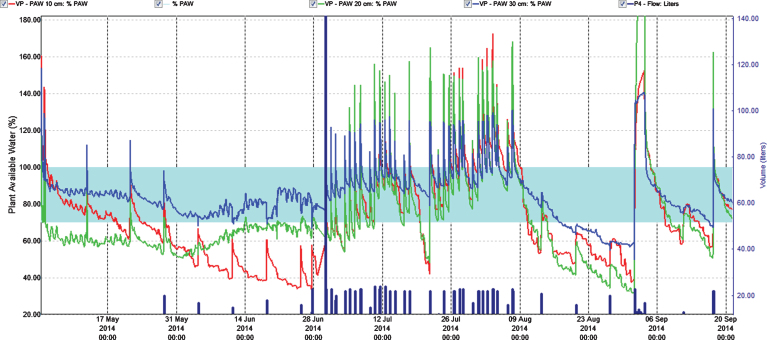
Percentage plant available water at three soil depths (Red = 10 cm, Green = 20 cm, Blue = 30 cm) in the first harvest year (2014). Easily available water (70–100%) is within the turkis zone. 100% available water = FC.

**Fig.5:**
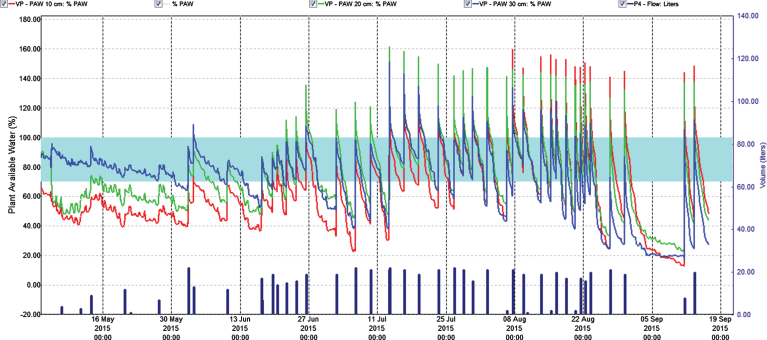
Percentage plant available water at three soil depths (Red = 10 cm, Green = 20 cm, Blue = 30 cm) in the second harvest year (2015). Easily available water (70–100%) is within the turkis zone. 100% available water = FC.

To control leaching we monitored water content in soil at three depths. This helped to adjust the fertilization program since water was the carrier of fertilizer. In the year of establishment the relation of the fertigation solution to the stock solution was 1 : 200 and water peaks at fertigation went high above FC (Field Capacity) (Figs. 1 and 3); we considered that a similar practice in 2014 would result in even higher water peaks since we increased length of watering pulses [17]. Therefore, we changed the relation to 1 : 100 in the two cropping years, and the peaks above FC the day of fertigation became acceptable in the growth and flowering stages, but still too high during harvest (Figs. 4 and 5). To avoid this in practice, we suggest fertigation twice a week with a shorter pulse, instead of once a week, to reduce the peaks. On a lighter soil than in our experiments adding fertilizer at every occasion of watering would be an option, but that would need a secure control of EC, and amount of water and fertilizer given as suggested by others [18]. In the establishing year, it seems like fertilizer equivalent to 1 g N m^–2^ s in balance with plant growth in a fertile soil. The experimental field was in open high polytunnel to avoid rainfall. In open field and especially on light soils, rainfall could wash out nutrient. Under such conditions, it is essential that the nutrient concentration in soil is monitored to make it possible to adjust the fertilization program.

**Fig.6:**
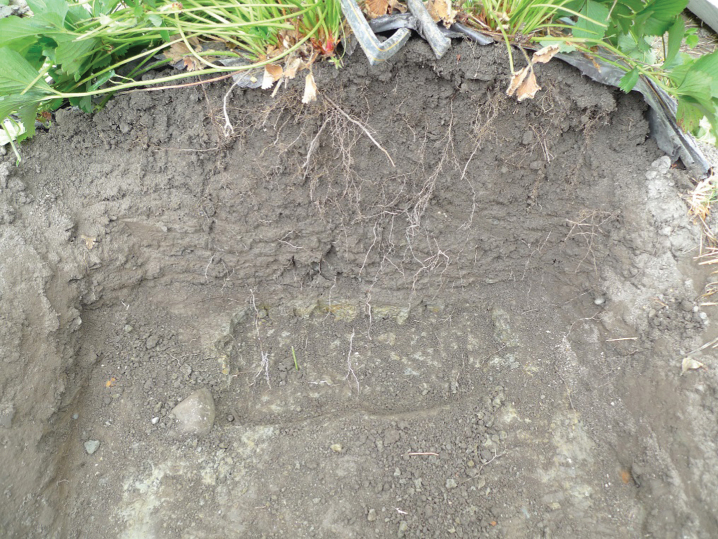
Cross section of a bed showing how strawberry roots distribute in the profile in August 2014 at fertilization level II. There were earthworm burrows, going deep into the plow layer.

In the first cropping year (2014) fertilization gradually increased from spring and throughout the harvest season and gradually decreased after harvest (Fig. 1). Using 3 g N m^–2^ (level II) during growth and flowering, did not alter nitrate and cation concentrations or EC in soil water much from the situation in July the previous year using 1 g N m^–2^ (Fig. 2, Tables 2 and 3). That could be because the plants were larger and had the capacity to use more nutrients, and that the temperature in 2014 was warmer than in 2013 enhancing respiration and uptake [17]. However, fertilization level III (6 g N m^–2^) was on the high side concerning concentration of nitrate at the flower stage. The good thing was that the concentration reduced from 15 to 25 cm depth, which also was the case for cations and EC (Fig. 2, Table 3). Plants can store large amounts of nitrate in their vacuoles and have in addition at least four different transport systems operating. Additionally many plant species can modify their root architecture to improve uptake and lateral roots will proliferate in nutrient rich soil patches [19]. We demonstrated in our experiments making a vertical cross cutting of one of the beds (using a spade), that roots concentrated just below the drip pipes seeking for water and nutrients (Fig. 6). There is not much information indicating what the nutrient level in soil should be in a strawberry field, but optimal mineral content in plant tissues is known [20], and it was shown that using a nutrient recipe for soilless culture (like we did) was not appropriate for fertilization in soil [8]. The situation in the field trial changed strongly moving into the harvest stage with a maximum EC of 0.25 for strongest fertilization. Control had relatively high levels of minerals compared to earlier development strages, but nitrate was close to zero probably because of a strong uptake. The reason for this could be high temperatures in July that year (average 19.7°C), with a potential to increase plant physiological processes and thereby uptake of nutrients, compared with a normal July with an average of 13.2°C [21]. It is obvious that the plants utilized the nutrients in the soil to provide for the generative development. At fertigation level III there is a positive gradient from 15 to 25 cm depth for Ca and Mg ions, but K ions decreased. The nitrate values were low for all fertilization levels indicating that the amount of fertilizer in the harvest period should be increased (Table 3, Fig. 2). The fertilization in autumn at fertilization level II (3 g N m^–2^) seems appropriate, while level III may be a little too high; although there is a negative gradient for all ions and EC from 15 to 25 cm depth. However, the total amount of fertilizer at strongest fertigation given for the whole season seem appropriate. It is generally a question of moving some of the fertilizer given during flowering (and early growth of unripe fruit) and in autumn into the harvest season.

In the second cropping year (2015) the sum of fertilizer given was equal to 2014, but experiences from 2014 made us change the fertigation program moving more of the fertilizer from the growth and flowering stage into the harvest season, compared with 2014. The fertilization stopped after the last harvest date 2 September (Fig. 1). EC was in some situations higher than recommended (0.51–1.50) for a not saline tolerant species like strawberry [15, 16]. In spite of the reduced use of fertilizer in the vegetative- and flowering-phase compared with 2014, the ion concentrations and EC were surprisingly high (Table 4, Fig. 2) in these stages. In the growth stage, concentration of nitrate at fertigation level III was close to 1400 mg l^–1^ at 15 cm depth. However, the roots were effective in taking up ions down to 25 cm lowering the concentration to 1000 mg l^–1^ (still high compared to a maximum of 200–350 ppm of nitrate, considered optimal for strawberry in a silt clay soil). For the other ions as well, there was a strong reduction in concentration at 25 cm soil depth compared to 15 cm. The high concentrations could be an influence of the large differences in temperature between the two years. The temperature averages in May, June and July 2014 and 2015 was 10.2, 13.0 and 19.6 and 8.4, 10.3 and 13.4, respectively [21], which would reduce the uptake of nutrients in 2015 because of low respiration, compared with the relative warm conditions in 2014 [22]. Additionally, evapotranspiration was higher in 2014 than in 2015, which would increase transport of ions into the plant lowering the levels in soil. However, we observed no visible injury on the above ground parts of the plants in our research, but EC close to 2 mS cm^–1^ at strongest fertilization during growth and flower development could give injury in saline sensitive plants like strawberry [14].

At fruit harvest, the ion concentration decreased for all fertigation levels except control compared with growth and flower stage, and it was generally higher than in 2014 as expected, probably because of a combination of changes in the fertilization scheme and the lower temperature (Fig. 1). The nitrate concentration differed little between fertilization levels II and III at 15 cm depth, but was lower at 25 cm depth for fertilization level II than for level III (Fig. 2). However, concentrations of cations and EC were higher at both depths for fertilization level III than for level II, but not considered too high and there was a decrease in concentrations down to 25 cm depth. What we learn from this is that we should have moved more of the nutrients given at growth and flower stage into the harvest stage. We therefore find that balanced fertigation equivalent to 6 g N m^–2^, giving 4 g N m^–2^ from two weeks before and during harvest is recommendable for a fruit yield of 3 kg m^–2^, as for ‘Sonata’ in 2015.

When watering in between the single days of weekly fertigation, the level of soil-ions decreased. Since we avoided high water peaks above field capacity, this practice should reduce leaching of nutrients. Therefore, it looks like the strawberry roots absorbed nearly all fertilizer given during harvest. As shown, roots are effective in taking up nutrients reducing the amount of ions in the soil profile between 15 and 25 cm. The roots developed well in the bed profile down to 35 cm and concentrated just below the drip pipes. A few roots moved even deeper into the hard clay layer (Fig. 6). This indicate that roots were active taking up nutrients down to at least 35 cm. However, in spite of roots going relatively deep the flux of water and fertilizer can be higher than the crop ability to absorb it, resulting in percolation and nutrient leaching.

#### 3.1.3 Nitrogen balance index (NBI) of strawberry leaves

**Fig.7:**
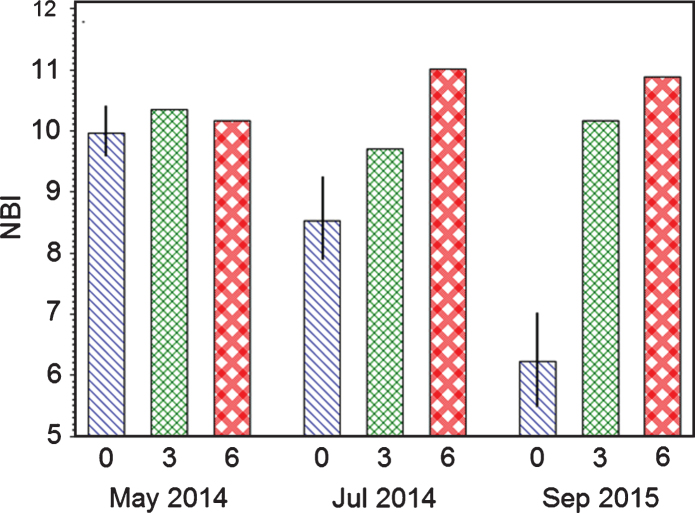
NBI index of strawberry leaves in two years influenced by three fertilization levels (Control = 0, II = 3 g N m^–2^, III = 6 g N m^–2^) as average of three cultivars.

NBI level was equal for all three fertilization levels May 2014 (Fig. 7). However, during harvest in July NBI of control (0) dropped strongly, while levels of fertigation level II and III were relatively unchanged, with a small reduction for level II and a small increase for level III. The next recordings were in September 2015 sampled after harvest, and it show that NBI for control was strongly reduced compared with the May and July recordings of the previous year; while the levels of fertilized plots had a similar appearance as the year before. The results correlate to the ion levels caused by differences in fertilization. It also indicate that optimum NBI level (average of adaxial and abaxial side) for strawberry is between 9.5 and 11.5, lower than for Blackcurrant and sourcherry [11].

### 3.2 Effect of cultivar

There was no significant difference in fruit yield and fruit weight between cultivars in 2014. ‘Sonata’ had the highest percentage of fruits larger than 25 mm diameter followed by ‘Florence’ and ‘Korona’. ‘Korona’ had more small fruits than ‘Sonata’ and more rotten fruit than ‘Sonata’ and ‘Florence’. There was no mildew infected or misshapen fruits recorded in 2014 (Table 5). In 2015 ‘Florence’ and ‘Sonata’ had larger fruits than ‘Korona’, and ‘Florence’ had higher total yield than the other cultivars, though the total yields of both ‘Korona’ and ‘Sonata’ must be considered as high, compared with historical data at our research station [4, 14]. ‘Korona’, however, was presenting badly, mainly because it was strongly mildew infested, had some rot and misshapen fruits and small fruit size. Due to the high amount of fruits smaller than 25 mm, only 58.8 percent of the fruit was marketable fruit. Contrasting, ‘Sonata’ yielded 88.5 and ‘Florence’ 81.4 percentage marketable fruit. The poor presentation of ‘Korona’ in 2015 colors the average of the two fruiting years, where ‘Korona’ is the loosing part compared with the two other cultivars for most tabled characters. In 2015 ‘Korona’ had 1.32 and 2.00 times as many harvested fruits, respectively, as ‘Florence’ and ‘Sonata’. In spite of a fruit number almost as high as ‘Korona’, ‘Florence’ gave a good yield in opposition to ‘Korona’. The high number of flowers is a result of optimal temperatures for flower bud development in late autumn 2014. ‘Sonata’ developed fewer fruits than ‘Korona’, but much better developed flower buds and much larger fruits. The mildew infestation of ‘Korona’ and ‘Florence’ increased at strongest fertilization, because we wanted to evaluate the effect of fertilization treatment and did not take any actions against mildew. However, the mildew infestation can easily be avoided [23].

**Table 5:** Effect of cultivar (Cv) on fruit weight (Fw) in g fruit^–1^, total fruit yield (T) in g m^–2^, percentage fruit yield in different fruit sizes (P),rot and mildew infection and rest, in two harvest seasons as average of three fertilization levels

Cv	Year	Fw	Fruit yield							
			T	*P*≥35	P30–35	P25–30	*P* < 25	Rot	Mild	Rest
Korona	2014	15.2	1.426	48.9	26.4	14.4	4.7	4.2	0	0
Florence		15.2	1.746	58.4	23.1	11.3	4.0	1.8	0	0
Sonata		16.6	1.331	62.3	22.6	8.6	1.9	2.5	0	0
Mean		15.7	1.568	56.5	24.0	11.4	3.5	2.8	0	0
Se		0.9^ns^	0.194^ns^	3.6^**^	1.7^ns^	2.1^*^	0.5^**^	1.0^*^	.	.
Korona	2015	13.3	2.906	20.3	19.9	18.6	12.8	8.7	15.4	2.2
Florence		19.3	3.932	48.8	19.5	13.1	4.6	6.6	5.6	0.9
Sonata		18.9	2.546	53.0	24.3	11.2	3.2	3.6	0.9	0.5
Mean		17.1	3.150	40.2	21.1	14.4	6.9	6.4	7.6	1.2
Se		0.9^***^	0.348^**^	2.7^***^	2.0^ns^	1.6^***^	0.8^***^	1.3^**^	4.7^*^	0,5^**^
Korona	Mean	14.2	2.207	34.6	23.2	16.4	8.8	6.5	7.8	1.1
Florence		17.3	2.866	53.6	21.3	12.2	4.3	4.2	2.8	0.5
Sonata		17.7	1.937	57.9	23.4	9.8	2.5	3.0	0.4	0.2
Mean		16.4	2.344	48.5	22.6	12.9	5.3	4.6	3.7	0.6
Se		0.6^**^	0.196^***^*	2.3^***^	1.3^*^	1.3^*^	0.5^***^	0.9^***^	2.4^***^	0.2^***^

^*,**,***,ns^indicate significant differences, respectively at levels of 5%, 1%, 0.1% and no significance.

## 4 Conclusion

The intentions of the experiments was to reduce nutrient leaching by establishing a practice where the ion levels in soil water decreased while the water moved downwards in the soil profile, and to adapt fertilization to the growing stages of the strawberry plant to optimize fruit yield. This was achieved by fertilizing before planting in the establishing year with 50 g m^–2^ of YaraMila Fullgjødsel 12-4-18 micro™ (YARA Norway). However, to give a small amount of a balanced nutrient solution in the first half of September in addition is recommendable [24]. In the harvesting years the best results was achieved using 6 g N m-2 in a balanced nutrient solution consisting of two nutrient stock solutions, one of 7.5 kg YaraLiva™ Calcinit per 100 l of water and a second of 7.5–9.0 kg per l of Kristalon™ Indigo per 100 l of water, with highest level of Kristalon during harvest. However, if fruit yield is 3 kg m^–2^ then should 4 g m^–2^ of the fertilizer be given from two weeks before harvest and during harvest; and at least two fertilization events per week is recommendable instead of the one that we used. Of the cultivars, ‘Sonata’ was best suited for growing in a high open polytunnel, but also ‘Florence’ gave a good impression except for a slight infestation of mildew in the second harvesting year and had the highest yield. In the second harvesting year ‘Korona’ fruits were strongly infested by mildew and developed very small fruits and low marketable yield.

## Conflict of interest

None to report.
